# Hypoxia Selects Bortezomib-Resistant Stem Cells of Chronic Myeloid Leukemia

**DOI:** 10.1371/journal.pone.0017008

**Published:** 2011-02-11

**Authors:** Michele Tanturli, Serena Giuntoli, Valentina Barbetti, Elisabetta Rovida, Persio Dello Sbarba

**Affiliations:** Dipartimento di Patologia e Oncologia Sperimentali, Università degli Studi di Firenze, and Istituto Toscano Tumori, Firenze, Italy; Roswell Park Cancer Institute, United States of America

## Abstract

We previously demonstrated that severe hypoxia inhibits growth of Chronic Myeloid Leukemia (CML) cells and selects stem cells where BCR/Abl_protein_ is suppressed, although mRNA is not, so that hypoxia-selected stem cells, while remaining leukemic, are independent of BCR/Abl signaling and thereby refractory to Imatinib-mesylate. The main target of this study was to address the effects of the proteasome inhibitor Bortezomib (BZ) on the maintenance of stem or progenitor cells in hypoxic primary cultures (LC1), by determining the capacity of LC1 cells to repopulate normoxic secondary cultures (LC2) and the kinetics of this repopulation. Unselected K562 cells from day-2 hypoxic LC1 repopulated LC2 with rapid, progenitor-type kinetics; this repopulation was suppressed by BZ addition to LC1 at time 0, but completely resistant to day-1 BZ, indicating that progenitors require some time to adapt to stand hypoxia. K562 cells selected in hypoxic day-7 LC1 repopulated LC2 with stem-type kinetics, which was largely resistant to BZ added at either time 0 or day 1, indicating that hypoxia-selectable stem cells are BZ-resistant *per se*, *i.e.* before their selection. Furthermore, these cells were completely resistant to day-6 BZ, *i.e.* after selection. On the other hand, hypoxia-selected stem cells from CD34-positive cells of blast-crisis CML patients appeared completely resistant to either time-0 or day-1 BZ. To exploit *in vitro* the capacity of CML cells to adapt to hypoxia enabled to detect a subset of BZ-resistant leukemia stem cells, a finding of particular relevance in light of the fact that our experimental system mimics the physiologically hypoxic environment of bone marrow niches where leukemia stem cells most likely home and sustain minimal residual disease *in vivo*. This suggests the use of BZ as an enhanced strategy to control CML. in particular to prevent relapse of disease, to be considered with caution and to need further deepening.

## Introduction

We previously demonstrated that severe hypoxia inhibits growth of leukemia cell populations and selects cells exhibiting properties of leukemia stem cells (LSC) [1]–[3]. Incubation of Chronic Myeloid Leukemia (CML) cells in hypoxia, including K562 as well as the corresponding primary cells (from blast-crisis patients), results in the complete suppression of BCR/Abl protein, but not mRNA, so that hypoxia-selected CML stem cells, while remaining genotypically leukemic, are phenotypically independent of BCR/Abl signaling and thereby refractory to the treatment with Imatinib-mesylate (IM) [1], [3]. This is very well in keeping with the notions that IM, despite its impressive efficacy as first-line therapy for patients with chronic phase CML, induces a state of minimal residual disease (MRD), rather than cure, and that LSC are the source of MRD. On this basis, we proposed that LSC responsible for MRD of CML home *in vivo* within severely hypoxic areas of bone marrow [3] where normal hematopoietic stem cells physiologically reside (the hypoxic stem cell niches) [4]–[7]. Thus, strategies to target IM-refractory LSC of CML are worth being explored within this context, taking advantage of LSC selection in hypoxia prior to drug administration *in vitro* to mimic the impact of treatment on cells already adapted to home in hypoxic tissue areas *in vivo*.

CML treatment with Bortezomib (BZ), a specific and reversible inhibitor of proteasome activity which is licensed for clinical use in mantle cell lymphoma and multiple myeloma, is being proposed to target LSC [8], on the basis of data indicating that BCR/Abl expression results in increased proteasome activity and that proteasome inhibition is cytotoxic against CML cell lines [9]. The study reported here addressed the effects of BZ on K562 as well as primary CML cells in severe hypoxia, to assess the sensitivity to BZ of hypoxia-selected LSC independently of, and in comparison with, that of unselected cells.

The quality of LSC selection, as well as the effects of BZ on hypoxia-selected LSC, were determined by the Culture-Repopulating Ability (CRA) assay, a simple and effective method to test *in vitro* the effects of drugs on LSC. This assay measures the stem cell potential in growth-inhibitory “selection” primary cultures (LC1) by determining the capacity of cells at the end of LC1 to repopulate growth-permissive “expansion” secondary cultures (LC2) and evaluating the kinetics of this repopulation. Further advantage to our experimental strategy is given by the fact that hypoxia selects LSC from every leukemia cell population tested so far, including clonal stabilized cell lines. The use of cell lines enables to work with relatively high numbers of hypoxia-selected cells and to reduce the complexity of system, as exogenous cytokines are necessary to support survival and growth of primary cells. Yet, data gathered with cell lines are of value for preclinical purposes, given their consistency with those obtained for primary cells and reported in this paper and earlier [3].

## Results

**Figure 1:**
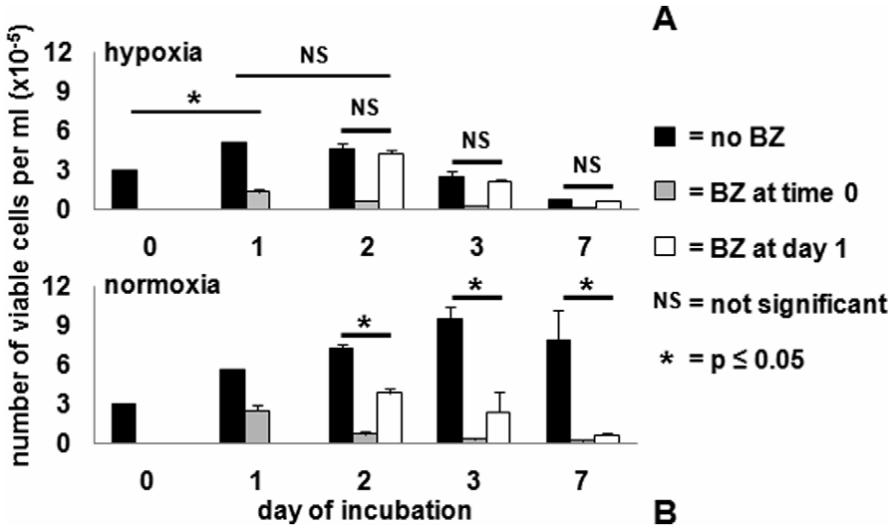
Effects of Bortezomib (BZ) on K562 cell bulk. Cultures were treated or not with a single dose of 0.5 nM BZ from time 0 to day 7 or from day 1 to day 7 and trypan blue-negative cells counted at the indicated times. Values represent means±S.E.M. of data from 3 independent experiments. (**A**): hypoxia (∼0.1% O_2_); (**B**): normoxia (21% O_2_).

K562 cell cultures were treated or not with a single dose of BZ from time 0 or day 1 to day 7. BZ addition to hypoxic cultures at time 0 markedly reduced the number of viable cells throughout incubation (Figure 1A), producing results comparable to those determined in normoxia (Figure 1B). On the contrary, BZ treatment at day 1 of hypoxic, but not normoxic, cultures was completely ineffective. It is worth pointing out that a 24-hour treatment with BZ in hypoxia yielded different results whether it was applied from time 0 to day 1 or from day 1 to day 2, an outcome indicating that a one-day pre-incubation in hypoxia protects cell bulk from the effects of BZ.

**Figure 2:**
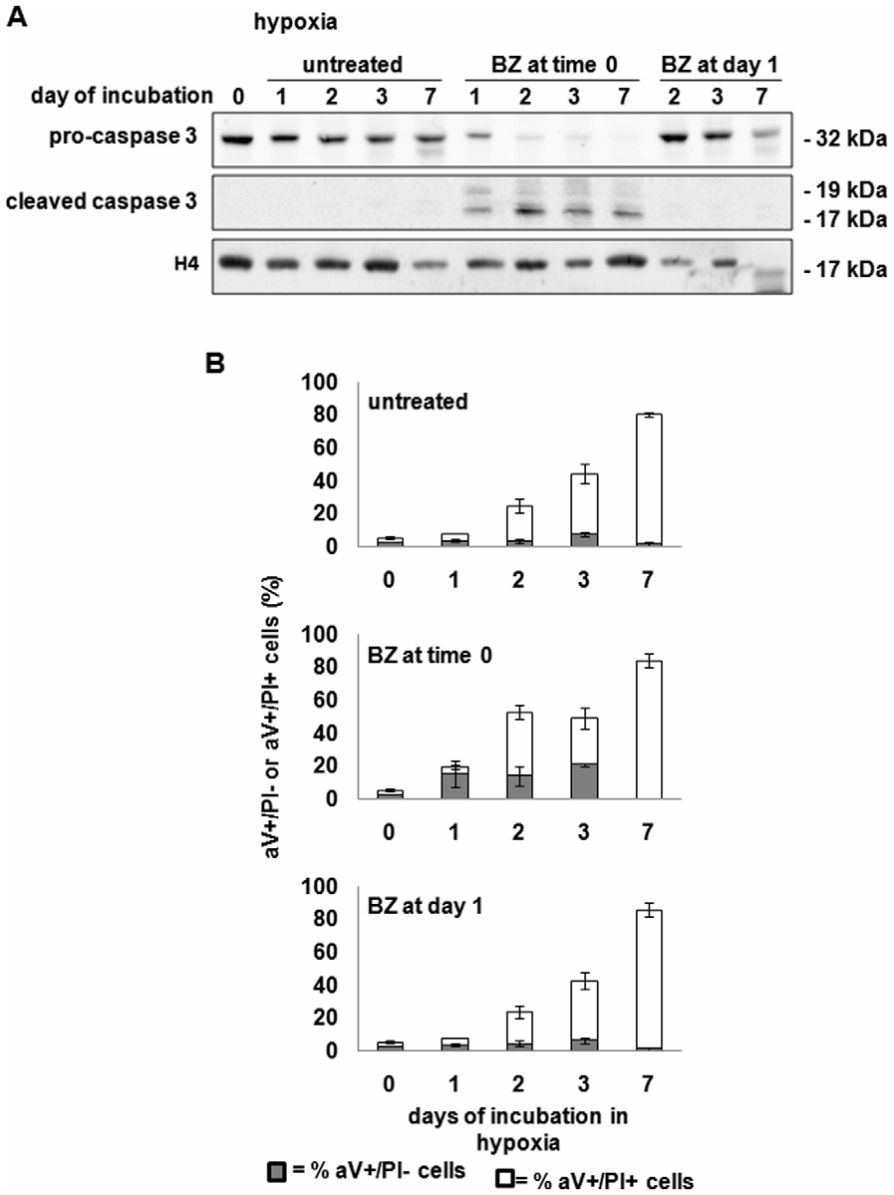
Induction of apoptosis by BZ in hypoxia. (**A**) Total cell lysates in Laemmli buffer were subjected to immuno-blotting with the indicated antibodies. Anti-H4 was used to verify equalization of protein loading. One representative experiment out of 3 is shown. (**B**) Percentages cells undergone “early” (annexin-V+/PI-) or “late” (annexin-V+/PI+) apoptosis, as determined by flow-cytometry. Values are means±S.E.M. of 3 independent experiments.

The hypoxia-dependent protection from the effects of BZ was confirmed by the marked early activation of caspase-3 and induction of apoptosis which followed BZ addition at time 0, but not day 1 (Figure 2), suggesting that hypoxia interfered with BZ by preventing caspase-dependent apoptosis of cell bulk [10]. At later incubation times, as expected [2], [3], hypoxia *per se* induced apoptosis in most cells.

**Figure 3:**
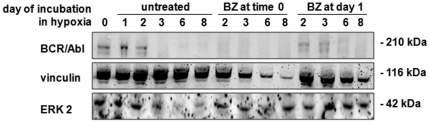
Effects of BZ on BCR/Abl protein expression in hypoxia. Total cell lysates in Laemmli buffer were subjected to immuno-blotting with an anti-Abl antibody. Anti-vinculin and anti-ERK1/2 antibodies were used to verify equalization of protein loading. One representative experiment out of 3 is shown.

Figure 3 shows that time-0 BZ markedly accelerated BCR/Abl_protein_ suppression occurring in untreated hypoxic cultures [1], [3], where BCR/Abl_protein_ was still well expressed at day 2. BZ treatment at day 1 failed to determine such an effect, indicating that a one-day incubation in hypoxia protected BCR/Abl_protein_ from BZ-induced suppression. When compared to time-0 BZ, day-1 BZ was not just ineffective, but actually delayed BCR/Abl_protein_ suppression, indicating that hypoxia suppressed BCR/Abl_protein_ at least in part via proteasoma.

**Figure 4:**
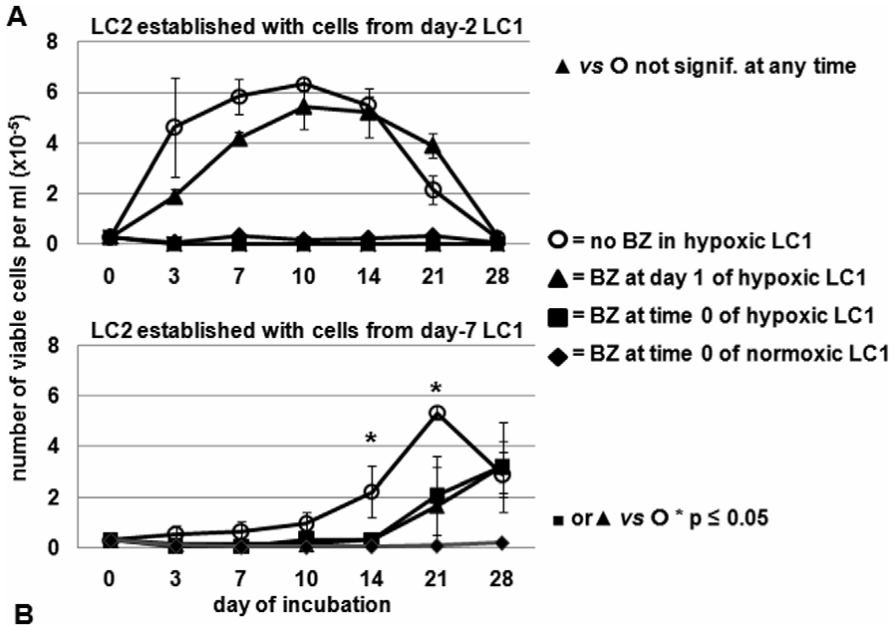
Effects of BZ on hypoxia-resistant K562 cell subsets. Cells treated as indicated in hypoxic or normoxic LC1 (established at 3×10^4^ viable cells/ml) were transferred at day 2 (**A**) or day 7 (**B**) into normoxic LC2 (3×10^4^ viable cells/ml) and trypan blue-negative cells counted at the indicated times of incubation in LC2. Values represent means±S.E.M. of data from 3 independent experiments.

The effects of BZ in hypoxia or normoxia on leukemia progenitor and stem cells were then determined by the CRA assay [1]–[3], [11]. Cells treated or not with BZ in hypoxic LC1 were transferred at different times of incubation therein into BZ-free normoxic LC2 to determine the pattern of their repopulation. Cells replated from day-2 hypoxic LC1, where hypoxia-dependent cell selection had not occurred yet and BCR/Abl_protein_ was still expressed [1], [3], rapidly repopulated LC2, to peak at day 10, as an effect of BCR/Abl-dependent growth stimulation (Figure 4A). BZ addition to hypoxic LC1 at day 1 did not alter this kinetics significantly, in keeping with the fact that day-1 BZ did not suppress BCR/Abl_protein_ (see Figure 3). BZ addition to hypoxic or normoxic LC1 at time 0 (or to normoxic LC1 at day 1; data not shown) abolished LC2 repopulation.

**Figure 5:**
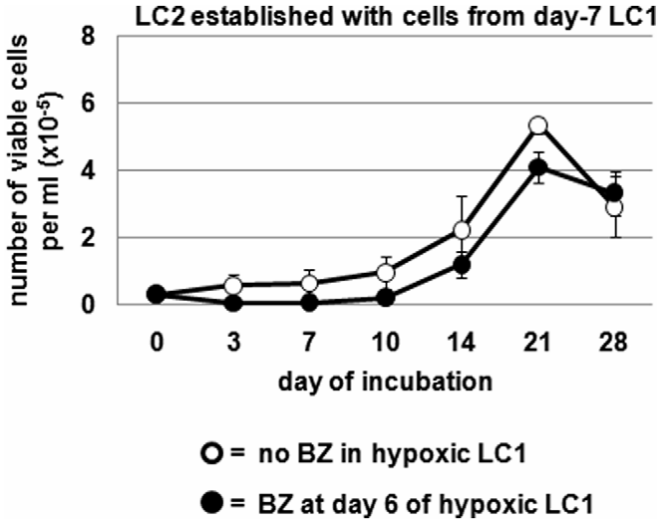
Effects of BZ on hypoxia-selected K562 cells. Cells from day-7 hypoxic LC1 treated with BZ at day 6 as indicated were transferred into normoxic LC2 (3×10^4^ viable cells/ml) and trypan blue-negative cells counted at the indicated times of incubation in LC2. Values represent means±S.E.M. of data from 3 independent experiments.

On the other hand, cell transfer to LC2 from day-7 LC1, *i.e.* following a one-log reduction of cell number in LC1 and the relative enrichment of BCR/Abl_protein_-negative cells (see Figures 1A and 3) [1], [3], resulted in a delayed LC2 repopulation, which reached its peak at day 21 (Figure 4B). Such a kinetics typically reflects the content of transplanted LC1 cells with LSC [2], [3], as well as normal hematopoietic stem cells [11], [12]. LC2 repopulation was significantly reduced, but not abolished, by BZ addition to hypoxic LC1 at either time 0 or day 1. BZ addition to normoxic LC1 at time 0 **(or day 1; data not shown)** abolished LC2 repopulation. The identical effects of time-0 and day-1 BZ administered in hypoxia indicated that LSC are in part capable *per se* (*i.e.* before their adaptation to, and enrichment in, hypoxia) to stand BZ action. To exploit such a resistance, however, LSC are required to be maintained in hypoxia, as BZ addition to normoxic LC1 abolished LC2 repopulation by either day-7 or day-2 LC1 cells. On the other hand, when hypoxic LC1 were treated with BZ at day 6 (*i.e.* after cell adaptation to, and enrichment in, hypoxia), LC2 repopulation resulted completely drug-insensitive (Figure 5). This indicated that LSC, once decisively selected in hypoxia, are completely resistant to BZ.

While we were completing our study, the effects of higher BZ doses, although for shorter times than in our experiments, were published [8]. When we tested, on this basis, the effects of the addition of 5–20 nM BZ to hypoxic LC1 at day 1, BZ was completely ineffective in either LC1 or LC2 (data not shown), indicating that a 24-hour pre-incubation in hypoxia confers resistant also to high-dose BZ.

**Figure 6:**
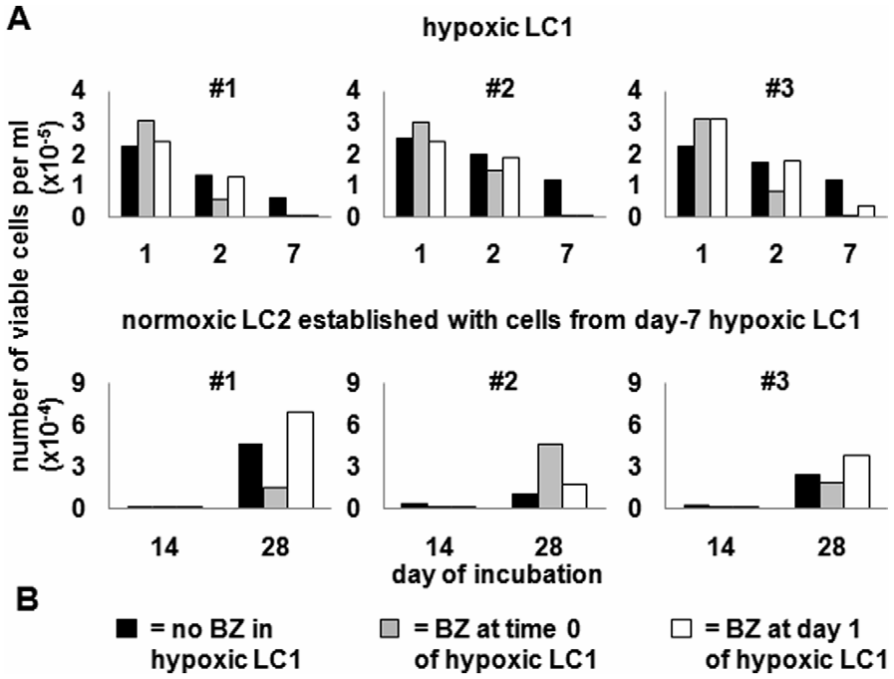
Effects of BZ on hypoxia-resistant primary CML cells. Immunomagnetically-selected CD34+ bone marrow cells from 3 blast-crisis CML patients were treated in hypoxic LC1 (established at 3×10^4^ viable cells/ml) as indicated (**A**) and transferred at day 7 into normoxic LC2 (3×10^4^ viable cells/ml) (**B**), where trypan blue-negative cells were counted at the indicated times of incubation. Values represent means±S.E.M. of data from 3 independent experiments.

The K562 data reported above were confirmed using CD34+ bone marrow cells explanted from blast-crisis CML patients. BZ enhanced the hypoxia-induced time-dependent reduction of the number of viable cells in LC1, irrespective of whether had been added at time 0 or day 1 (Figure 6A), the effect of time-0 BZ being perhaps faster (see day 2). CML cells from day-7 hypoxic LC1 repopulated normoxic LC2 reaching peak after 4 weeks of incubation. LC2 repopulation was not consistently affected by BZ treatment of LC1 at either time 0 or day 1 (Figure 6B). LC2 repopulation was paralleled by re-expression of BCR/Abl_protein_ (data not shown), as expected [1], [3]. Thus, primary CML cells were found to contain hypoxia-selectable LSC completely resistant to BZ.

## Discussion

This study showed, in primary as well as K562 CML cells, differences with respect to response to BZ between cell bulk (sensitive) and a cell subset containing LSC selected following prolonged incubation in hypoxia (resistant). Two different ways for CML cells to adapt to hypoxia were also evidenced: a “rapid adaptation” (occurring within 1 day of incubation in hypoxia) of cell bulk emerged, capable to prevent BZ-induced caspase activation, apoptosis and BCR/Abl_protein_ suppression, and the consequent destruction of culture. Such a rapid adaptation to hypoxia was not necessary for a minority of cells suitable to survive independently of BCR/Abl_protein_ expression and thereby undergo selection through 7 days of incubation in hypoxia. These cells were in large part BZ-insensitive *during* their selection irrespective of time (time 0 or day 1) of drug addition (see Figure 4B), and completely insensitive to BZ treatment *after* cell selection in hypoxia (at day 6; Figure 5). Thus, a leukemia cell subset exists which is predisposed to exhibit a stem cell phenotype and to escape hypoxia-induced apoptosis, so that it can then undergo a relatively slow program of adaptation to hypoxia, which progressively consolidates BZ resistance.

The most relevant result of this study is that hypoxia-adapted LSC of blast-crisis CML are completely resistant to BZ. This resistance, if one assumes hypoxia-adapted LSC to be a subset of total LSC, is likely to explain why BZ was found to determine a marked decline of BCR/Abl-positive chronic-phase LSC, but not their eradication [8]. We believe the hypoxia-adapted LSC subset to be the main source of MRD, as it is adapted to home in the physiologically hypoxic stem cell niches of bone marrow [4]–[7], [13]. Under this perspective, the more interesting results reported here are those obtained following BZ treatment at day 1 (as well as day 6) of incubation in hypoxia, as they mimic more realistically what occurs when the drug administered *in vivo* encounters LSC already placed in hypoxic tissue areas.

The demonstration that hypoxia-selected LSC of CML, which we previously showed to be refractory to IM [1], [3], are also resistant to BZ needs to be discussed in view of the possible development of a BZ/IM combination protocol for treatment of CML. Our results indicated that there is no point in using BZ to target hypoxia-selected, BCR/Abl_protein_–negative LSC. On the other hand, we also showed (see above) that K562 cell bulk can acquire resistance to BZ via a rapid program of adaptation to hypoxia. As this occurs while cells are still IM-sensitive due to the maintained expression of BCR/Abl_protein_, there is no apparent advantage either for using BZ together with IM to target BCR/Abl_protein_–expressing cells. Taken together, our results suggest the use of BZ as an enhanced strategy to control CML to be considered with caution and to need further deepening, especially considering the known toxic effects of BZ, including myelosuppression [8].

## Materials and Methods

### Cells and culture conditions

K562 and primary CML cells were routinely cultured in RPMI 1640 medium supplemented with 10% foetal bovine serum, 50 units/ml penicillin and 50 µg/ml streptomycin (all from EuroClone, Paington, UK, E.U.). Primary CML cells were collected after informed consent to the use for basic *in vitro* research, according to the Helsinki declaration and following approval of the Ethics Committee, at the Division of Hematology of Università di Firenze. CD34-positive cells were cultured in the presence of Flt-3 ligand (50 ng/ml) TPo (20 ng/ml) and SCF/KL (50 ng/ml) in LC1 and of SCF (50 ng/ml), G-CSF (100 ng/ml), IL-6 (20 ng/ml) and IL3 (10 ng/ml) in LC2 (all from PeproTech, Rocky Hill, NJ, U.S.A.). Exponentially growing cells were plated at 3×10^5^/ml and incubated in hypoxia (∼0.1% O_2_) in a Concept 400 anaerobic incubator (Ruskinn Technology Ltd., Bridgend, UK, E.U.) or normoxia (21% O_2_), at 37°C in a water-saturated atmosphere containing 0.1% O_2_, 94.9% N_2_ and 5% CO_2_.

### Reagents

Bortezomib (Velcade®, Millennium Pharmaceuticals, Cambridge, MA, U.S.A.) was added to cultures at 0.5 nM (cell lines and primary cells) at the indicated times.

### Measurement of cell viability and apoptosis

Viable cells were counted in a hemocytometer by trypan blue exclusion. Apoptotic cells were detected by using a FACSCanto flow-cytometer (Becton & Dickinson, Franklin Lakes, NJ, U.S.A.), after staining with FITC-conjugated annexin V and propidium iodide (PI), using the Annexin V-fluos staining kit (Roche Diagnostics, Basel, Switzerland) and following manufacturer's instructions. The percentages of annexin-V+/PI- or annexin-V+/PI+ cells are considered to reflect “early” or “late” apoptosis.

#### The CRA assay

This assay estimates the culture-repopulating power of normal [11], [12] or leukemic [1]–[3] hematopoietic cells undergone a selection treatment (e.g. hypoxia) in a primary liquid culture (LC1) by means of their transfer in fresh medium to non-selective conditions (e.g. normoxia) in a growth-permissive secondary culture (LC2) and the measure of its repopulation following a further incubation therein. Cell subsets rescued from LC1 at different times repopulate LC2 with different kinetics, the time necessary to reach the peak of LC2 repopulation reflecting the hierarchical level of stem/progenitor cells enriched in LC1. The CRA assay is a non-clonogenic assay capable to reveal *in vitro* stem cells endowed with marrow-repopulating ability *in vivo* [11]. The adaptation of CRA assay to leukemia cell populations enabled to detect different subsets of leukemia stem or progenitor cells [1]–[3].

### Protein separation and detection

Cells (5×10^6^) were washed twice with ice-cold PBS containing Na_3_VO_4_ 100 µM and solubilyzed by incubating for 10 min at 95°C in Laemmli buffer (Tris/HCl 62.5 mM, pH 6.8, 10% glycerol, 0.005% blue bromophenol, 2% SDS). Lysates were clarified by centrifugation (20000 g, 10 minutes, RT) and protein concentration in supernatants was determined by the BCA method. Aliquots (30 µg/sample) were boiled for 10 min in the presence of 100 mM 2-mercaptoethanol, subjected to SDS-PAGE in 7.6% polyacrylamide minigels and then transferred onto PVDF membranes (Millipore Corporate, Billerica, MA, U.S.A.) by electroblotting. Membranes were blocked in Odyssey Blocking Buffer (OBB)/PBS (1∶1) for 1 hour at RT and incubated in a 1∶1000 dilution of antibody in PBS 0,1% Tween (T-PBS)/OBB (1∶1) overnight at 4°C. Primary (all rabbit) antibodies were: anti-c-Abl and anti-cleaved-caspase 3 (from Cell Signaling Technology, Danvers, MA, U.S.A.), anti-caspase 3 and anti-ERK-1/2 (from Santa Cruz Biotechnology, S. Cruz, CA, U.S.A.), anti-H4 (from Millipore, Billerica, MA, U.S.A.) and anti-vinculin (from Sigma-Aldrich®, St. Louis, MO, U.S.A.). Secondary anti-IgG antibodies were IRDye®800CW- or IRDye®680-conjugated (LI-COR® Biosciences, Lincoln, NE, U.S.A.). Antibody-coated protein bands were visualized by the Odyssey Infrared Imaging System Densitometry (LI-COR®).

### Statistical Analysis

All experiments were performed in triplicate or higher numbers of repeats. Statistical significance was evaluated by Student's *t*-test for paired samples; p values <.05 (two-sided) were considered statistically significant.

## References

[1] Giuntoli S, Rovida E, Barbetti V, Cipolleschi MG, Olivotto M (2006). Hypoxia suppresses BCR/Abl and selects imatinib-insensitive progenitors within clonal CML populations.. Leukemia.

[2] Giuntoli S, Rovida E, Gozzini A, Barbetti V, Cipolleschi MG (2007). Severe hypoxia defines heterogeneity and selects highly immature progenitors within clonal erythroleukemia cells.. Stem Cells.

[3] Giuntoli S, Tanturli M, Di Gesualdo F, Barbetti V, Rovida E (2011). Glucose availability in hypoxia regulates the selection of Chronic Myeloid Leukaemia progenitor subsets with different resistance to Imatinib-mesylate.. Haematologica.

[4] Dello Sbarba P, Cipolleschi MG, Olivotto M (1987). Hemopoietic progenitor cells are sensitive to the cytostatic effect of pyruvate.. Exp Hematol.

[5] Cipolleschi MG, Dello Sbarba P, Olivotto M (1993). The role of hypoxia in the maintenance of hematopoietic stem cells.. Blood.

[6] Parmar K, Mauch P, Vergilio JA, Sackstein R, Down JD (2007). Distribution of hematopoietic stem cells in the bone marrow according to regional hypoxia.. Proc Natl Acad Sci USA.

[7] Eliasson P, Jönsson JI (2010). The hematopoietic stem cell niche: low in oxygen but a nice place to be.. J Cell Physiol.

[8] Heaney NB, Pellicano F, Zhang B, Crawford L, Chu S (2010). Bortezomib induces apoptosis in primitive chronic myeloid leukemia cells including LTC-IC and NOD/SCID repopulating cells.. Blood.

[9] Jagani Z, Song K, Kutok JL, Dewar MR, Melet A (2009). Proteasome inhibition causes regression of leukemia and abrogates BCR-ABL-induced evasion of apoptosis in part through regulation of Forkhead tumor suppressors.. Cancer Res.

[10] Albero MP, Vaquer JM, Andreu, EJ, Villanueva JJ, Franch L (2010). Bortezomib decreases Rb phosphorylation and induces caspase-dependent apoptosis in Imatinib-sensitive and -resistant Bcr-Abl1-expressing cells.. Oncogene.

[11] Cipolleschi MG, Rovida E, Ivanovic Z, Praloran V, Olivotto M (2000). The expansion of murine bone marrow cells preincubated in hypoxia as an in vitro indicator of their marrow-repopulating ability.. Leukemia.

[12] Ivanović Z, Belloc F, Faucher JL, Cipolleschi MG, Praloran V (2002). Hypoxia maintains and interleukin-3 reduces the pre-colony-forming cell potential of dividing CD34(+) murine bone marrow cells.. Exp Hematol.

[13] Olivotto, M, Dello Sbarba P (2008). Environmental restrictions within tumor ecosystems select for a convergent, hypoxia-resistant phenotype of cancer stem cells.. Cell Cycle.

